# Emerging evidence for *CHFR* as a cancer biomarker: from tumor biology to precision medicine

**DOI:** 10.1007/s10555-013-9462-4

**Published:** 2013-12-28

**Authors:** Sarah Derks, Arjen H. G. Cleven, Veerle Melotte, Kim M. Smits, Johann C. Brandes, Nilofer Azad, Wim van Criekinge, Adriaan P. de Bruïne, James G. Herman, Manon van Engeland

**Affiliations:** 1 Department of Medical Oncology, Cancer Center Amsterdam, VU University Medical Center, Amsterdam, The Netherlands; 2 Department of Pathology, GROW-School for Oncology and Developmental Biology, Maastricht University Medical Center, 6200 MD Maastricht, The Netherlands; 3 Department of Radiation Oncology (MAASTRO Clinic), GROW-School for Oncology and Developmental Biology, Maastricht University Medical Center, Maastricht, The Netherlands; 4 Department of Hematology and Oncology, Atlanta VA Medical Center Winship Cancer Institute, Emory University, Atlanta, GA USA; 5 Department of Mathematical Modelling, Statistics and Bioinformatics, Ghent University, Ghent, Belgium; 6 MDxHealth, Irvine, CA USA; 7 Department of Gastrointestinal Oncology, The Sidney Kimmel Comprehensive Cancer Center at the Johns Hopkins University School of Medicine, Baltimore, MD USA; 8 Department of Tumor Biology, The Sidney Kimmel Comprehensive Cancer Center at the Johns Hopkins University School of Medicine, Baltimore, MD USA

**Keywords:** *CHFR* promoter methylation, Predictive biomarker, Taxane sensitivity

## Abstract

Novel insights in the biology of cancer have switched the paradigm of a “one-size-fits-all” cancer treatment to an individualized biology-driven treatment approach. In recent years, a diversity of biomarkers and targeted therapies has been discovered. Although these examples accentuate the promise of personalized cancer treatment, for most cancers and cancer subgroups no biomarkers and effective targeted therapy are available. The great majority of patients still receive unselected standard therapies with no use of their individual molecular characteristics. Better knowledge about the underlying tumor biology will lead the way toward personalized cancer treatment. In this review, we summarize the evidence for a promising cancer biomarker: checkpoint with forkhead and ring finger domains (*CHFR*). *CHFR* is a mitotic checkpoint and tumor suppressor gene, which is inactivated in a diverse group of solid malignancies, mostly by promoter CpG island methylation. *CHFR* inactivation has shown to be an indicator of poor prognosis and sensitivity to taxane-based chemotherapy. Here we summarize the current knowledge of altered *CHFR* expression in cancer, the impact on tumor biology and implications for personalized cancer treatment.

## Introduction

Over the last 20 years, there has been a revolution in the perspective of cancer treatment. Improvement of molecular profiling techniques such as next generation sequencing and whole genome methylation analysis made it possible to compare thousands of molecules simultaneously with high accuracy and speed. These studies have allowed novel and meaningful insights in the biology of cancer. Lung, breast, colorectal and many other cancers have shown to be heterogeneous diseases, which develop through specific molecular alterations that influence the clinical presentation, prognosis and response to therapy [1]. The diversity of molecular background and resultant biological behavior can be harnessed into an individualized biology-driven treatment, instead of the present “one-size-fits-all” approach. For some cancers, personalized cancer treatment is already implemented in daily practice.

In breast cancer treatment, for instance, it is now standard to test and target increased human epidermal growth factor receptor 2 (HER2) with both monoclonal antibodies such as trastuzumab or small molecule inhibitors such as lapatinib [2]. In non-small cell lung cancer (NSCLC) patients, testing for mutations in *EGFR* and *KRAS*, and *EML4-*anaplastic lymphoma kinase (*ALK*) gene rearrangements to select appropriately targeted therapy occurs on a routine basis. Mutations in the kinase domain of *EGFR* have shown to be a strong predictor of response to EGFR tyrosine kinase inhibitors (TKIs) erlotinib and gefitinib [3]. These patients respond better to EGFR TKIs than to chemotherapeutic agents carboplatin/paclitaxel, reflected by a significantly improved progression-free survival [4]. Furthermore, patients with NSCLC harboring the ALK rearrangement, which occurs in about 7 % of NSCLCs, benefit from ALK inhibitor crizotinib [5]. A recent prospective randomized phase III study compared crizotinib therapy to chemotherapy, pemetrexed or docetaxel, in 347 locally advanced or metastatic *ALK*-positive lung cancers, and clearly showed that crizotinib therapy is associated with a higher response rate 65 % (95 % CI, 58 to 72) versus 20 % (95 % CI, 14 to 26) (*P* < 0.001) but also an improved quality of life compared to chemotherapy. The relatively low incidence of *EGFR* and *ALK* aberrations in non-Asian patients, however, account for the fact that ∼87 % of patients with NSCLC still receive conventional chemotherapy with no suitable biomarkers for therapy selection. The same accounts for women with triple-negative breast cancer who do not benefit from anti-hormonal therapy or trastuzumab and for whom effective treatment is limited [6].

Other examples of useful biomarkers are *KRAS* mutation testing to predict benefit from monoclonal antibodies against EGFR, cetuximab and panitumumab, in metastatic colon cancer [7] and *BRAF* V600E mutation analysis in metastatic melanoma in order to predict responsiveness to BRAF inhibitors such as vemurafenib [8]. Salient to this review, testing for promoter CpG island methylation of DNA repair gene O6-methylguanine-DNA methyltransferase, *MGMT*, guides the clinical management of glioblastoma. *MGMT* is able to reverse the damage acquired by alkylating agents and therefore promotes methylation, and subsequent silencing of *MGMT* is associated with increased progression-free and overall survival after therapy with alkylating agents such as temozolomide [9, 10].

Although these examples display the promise of personalized cancer treatment and more biomarkers are being discovered, work is still in progress. For most cancers and cancer subgroups, no biomarkers and effective targeted therapy are available and therefore the great majority of patients still receive standard therapies with no individualization based on their tumor’s molecular characteristics.

In this review, we highlight a promising novel biomarker for which multiple lines of evidence are emerging: *checkpoint with FHA and ring finger domains* (*CHFR). CHFR* is a mitotic checkpoint- and tumor suppressor gene and is inactivated in a diverse number of solid malignancies. *CHFR* is most frequently inactivated by promoter CpG island methylation and has shown to be a marker of poor prognosis and increased sensitivity to treatment with taxanes. Here we summarize literature on the relevance of altered *CHFR* expression in cancer.

## *CHFR*: an important regulator of cell cycle progression

*CHFR* is an early mitotic checkpoint gene that functions as a key player in controlling chromosomal integrity [11].

CHFR is expressed in the cytoplasm of all normal tissues and accumulates in the nucleus in response to microtubule poisoning or radiation damaging stress. After localization into the nucleus, CHFR becomes phosphorylated by protein kinase B (PKB/AKT), a member of the PI3K signaling pathway [12]. The nuclear distribution, mobility and function of CHFR are dependent upon interaction with promyelocytic leukemia protein (PML) bodies [13, 14]. CHFR expression levels fluctuate greatly during different stages of the cell cycle. Microtubule stress will lead to an elevation of CHFR expression levels and a mitotic arrest. To the contrary, auto-ubiquitination activity and degradation of CHFR, which is stimulated by AKT, are a prerequisite for mitotic entry [15]. Thereby, CHFR controls cell cycle progression at the G2/M transition as well.

It is not known how CHFR senses microtubule stress, but it has been shown that CHFR localizes to the mitotic spindle by an interaction with TCTP, a protein involved in microtubule stabilization and β-tubulin [16]. Disruption of the spindle causes CHFR to deliberate from TCTP and the mitotic spindle, which will enable the activation of signaling pathways and ultimately delay cell cycle progression [17].

These signaling pathways prevent entry into mitosis by inhibiting the activation of Cdc25 phosphatases that are able to activate the cyclin B1-Cdk1 kinase.

**Fig. 1:**
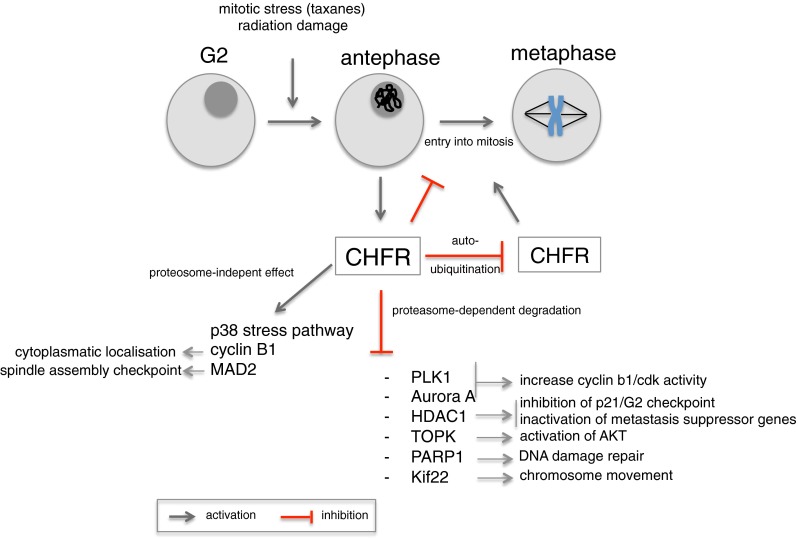
*CHFR* delays entry into metaphase in respons to microtubular stress by effecting target proteins in a proteosome-dependent and a proteosome-independent manner

CHFR is able to influence the mitotic checkpoint by a proteosomal-dependent and a proteosomal-independent mechanism (Fig. 1).

CHFR was first described to function as an E3 ubiquitin ligase, which ubiquitinates and targets proteins for degradation by the S26 proteosome [15, 18]. One of the target proteins is polo-like kinase 1 (PLK1). PLK1 is a serine/threonine kinase that is involved in the phosphorylation of Cdc25, thereby regulating cyclin B1-cdk activity. PLK1 phosphorylation and activation are established by another kinase, Aurora A [19–22]. By ubiquitination and degradation of both PLK1 and Aurora A, CHFR is able to inhibit the formation of the cyclin B1-Cdk complex and thereby promote cell cycle arrest [20, 23]. Although *in vitro* data are appealing, evidence that CHFR targets PLK1 for degradation *in vivo* as well is weak. There are conflicting studies that did not observe a decrease in PLK1 and Aurora A protein expression in response to microtubule poison [24–26]. Differences in study design and molecular environment make it difficult to compare results and therefore more studies are needed to clarify this inconsistency.

Other targets for ubiquitination and protein degradation by CHFR are chromokinesine protein Kif22[27], histone deacetylase HDAC1[28] and poly(ADP-ribose) 1 PARP1[29]. Kif22 plays a role in the organization of spindle microtubules and chromosome movement and regulation of Kif22 activity by CHFR is important for maintaining chromosomal stability [27]. HDAC1 is a histone deacetylase that is able to inhibit the expression of cell cycle genes such as p21. By ubiquitination of HDAC1, CHFR is able to reverse HDAC1-induced repression of p21 and thereby restore the p21-G1 checkpoint [28, 30, 31]. Interestingly, CHFR was also shown to inhibit invasiveness and metastatic potential caused by HDAC1 expression by the regulation of metastasis suppressors, KAI1 and E-cadherin [28].

PARP1 plays a role in the DNA damage response and is involved in the recruitment of CHFR to DNA damage sites immediately after DNA damage has occurred [29, 32]. *CHFR* then participates in a cascade of protein ubiquitination. One of the proteins that becomes ubiquitinated and degraded is PARP-1 itself. Thereby, CHFR is able to detach PARP-1 from the chromatin, which is an important step in the DNA damage repair response [29].

**Fig. 2:**
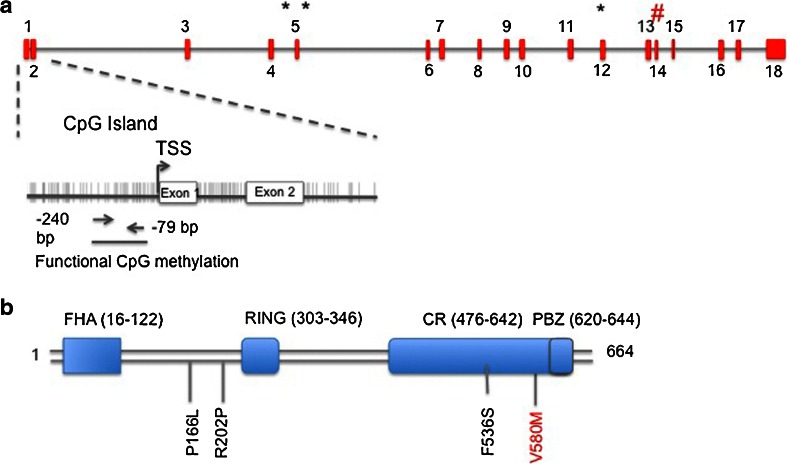
*CHFR* gene and protein. Schematic representation of promoter CpG island methylation, mutation and polymorphisms with functional significance. of **a** *CHFR* gene encompassing 18 exons. CpG island is enlarged with CpG dinucleotides as vertical lines. TSS: transcription start site * mutation. # polymorphism. **b** CHFR protein consisting of 664 aminoacids. FHA: forhead-associated domain. RING: ringfinger domain. CR: cysteine-rich domain. PBZ: RAR-binding zinc-finger domain. Mutations in *black*, polymorphism in *red*

CHFR binds to PARP-1 via the RAR-binding zinc finger domain, which is situated in the cysteine region of CHFR. As the name illustrates, CHFR contains a N-terminal FHA domain, a central RING finger domain, and a C-terminal cysteine-rich domain (Fig. 2). The function of the FHA domain is largely unknown but is required for the checkpoint function and might be involved in the binding to phosphorylated proteins [19]. The RING finger domain is important for the ubiquitinating activity of CHFR and is able to form lysine 48- and lysine 63-linked polyubiquitination chains [25]. The cysteine-rich domain is important for the interaction between CHFR and target proteins [19–22]. Inside the cysteine-rich region, the RAR-binding zinc-finger (PBZ) is situated which is able to bind poly(ADP-ribose)PARP family members such as PARP-1.

Recently, it was shown that CHFR also ubiquitinates and regulates the expression of TOPK [33]. TOPK is a promitotic serine/threonine kinase that phosphorylates and inactivates downstream substrate PTEN, which will lead to activation of AKT. By inhibiting TOPK, CHFR prevents the TOPK-induced activation of AKT and thereby blocks G2/M progression.

CHFR can also influence the mitotic checkpoint by functioning as an ubiquitin ligase that targets proteins not for degradation but for activation of signal transduction. By catalyzing the formation of noncanonical Lys63-linked polyubiquitin chains [25], CHFR was shown to activate the p38 stress kinase pathway, which will reverse chromosome condensation and induce a mitotic arrest [34]. Furthermore, CHFR indirectly inhibits the nuclear accumulation of cyclin B [24], thereby preventing the formation of the cyclin B1/cdk complex by the same mechanism, and also interacts with mitotic arrest deficient 2 (MAD2). MAD2 is a key protein in the spindle assembly checkpoint by its ability to sense improper spindle attachment and inhibit the anaphase-promoting-complex [35]. MAD2 is dependent upon binding to CHFR for its activation and transport to the kinetochore. In the absence of CHFR, MAD2 is not able to inhibit anaphase progression, which will result in mitotic defects [36].

Above-summarized data clearly show that *CHFR* is an important regulator of cell cycle progression. Since inactivation of CHFR promotes chromosomal defects and via activation of HDAC1 tissue invasion [28], CHFR malfunction is thought to play an important role in cancer progression and metastasis.

## *CHFR* inactivation in cancer and its role as tumor suppressor

*CHFR* is more frequently inactivated in cancer than all other mitotic checkpoint control genes together [11]. Scolnick et al. were the first to report lack of *CHFR* expression in neuroblastoma and colorectal cancer cell lines [11]. Absence of CHFR in these cell lines resulted in a high mitotic index when exposed to microtubule stress compared to wild-type cancer cells, which was restored by reintroduction of functional CHFR. In a breast cancer cell line model, decreased *CHFR* expression resulted in an accelerated growth rate, enhanced invasiveness and amplified colony formation.

In order to study the physiological role of CHFR and its function in tumorigenesis, Chfr knockout mice have been generated [20]. Chfr knockout mice develop invasive lymphomas and solid tumors (lung, liver, gastrointestinal) after 40 weeks and have an increased susceptibility to chemical carcinogenesis [20]. Embryonic fibroblasts from Chfr-deficient mice show substantial aneuploidy and polyploidy. Therefore, *CHFR* inactivation is expected to participate in the acquisition of chromosomal defects and a chromosomal instability phenotype in cancer. In primary colorectal and breast cancer tissue, however, *CHFR* inactivation is not associated with chromosomal instability [37]. In colorectal cancer (CRC) and gastric cancer, *CHFR* inactivation is associated, however, with microsatellite instability (MSI) and *MLH1* promoter CpG island methylation. The mechanism underlying the association between *CHFR* inactivation and MSI is unknown but might be due to an underlying DNA methylation defect that causes promoter CpG island methylation of both *CHFR* and *MLH1*. Murine studies, however, demonstrated that simultaneous loss of *Chfr* and *Mlh1* synergistically increased predisposition to cancer development, which implicates a more functional interaction [38].

Furthermore, a recent study shows an additional role for *CHFR* in regulating expression of pro-inflammatory chemokine interleukin-8 (IL-8). CHFR is able to inhibit the NFκB signaling pathway and IL-8, which subsequently resulted in decreased angiogenesis and cell migration [39–41]. Inactivation of *CHFR* triggers NFκB signaling activity and thereby accelerates angiogenesis and a metastatic phenotype, which is associated with a poor prognosis.

## Genetic and epigenetic mechanisms of *CHFR* transcriptional silencing

In the last decade, disrupted *CHFR* expression has been described in multiple cancer tissues (Table 1). Although promoter CpG island methylation is the most frequently occurring alteration leading to *CHFR* inactivation, genetic alterations have been observed occasionally. Scolnick and Halazonetis were the first to describe a sequence variation in the cysteine-rich domain of CHFR in osteosarcoma cell line U2OS [11] (Fig. 2). The variation consisted of a G to A transition leading to substitution of valine 539 by methionine and was initially interpreted as a missense mutation with functional impairment but turned out to be a polymorphism [42]. The relevance of variant genotypes was further studied in a series of 462 colorectal cancer patients and 245 healthy controls [43]. This study showed that the A allele of the GA variant was associated with a reduced CRC risk (*P* = 0.02; OR, 0.496; 95 % CI, 0.279–0.883). Thereby, it was shown that polymorphisms in the *CHFR* gene can be used as indicator for colorectal cancer susceptibility.

**Table 1:** *CHFR* inactivation in multiple cancers

Cancer	Aberration	Method, region analyzed	Percentage of methylation	Ref
Breast cancer	Reduced expression Methylation	IHC demethylation and northern blot	36 % (51/142) 8 % (2/24) (cell lines)	[70] [71]
Bladder cancer	Methylation	MLPA	18.7 % (17/91)	[72]
Colorectal cancer	Methylation	COBRA MSP, −281 to +51 bp COBRA MSP, −240 to −73 bp MSP, −226 to −82 bp qMSP, +221 to +325 bp MSP, −240 to −73 bp	40 % (25/63) 37 % (11/30) 53 % (27/51) (adenomas) 41 % (29/71) 26% (25/98) 24 % (217/888) 31 % (19/61)	[73] [74] [71] [75] [76] [77] [67]
Gastric cancer	Methylation	COBRA MSP, −9 to +98 bp COBRA MSP, −163 to −8 bp	39 % (24/61) 35 % (25/71) 44 % (19/43) 52 % (24/46)	[56] [78] [79] [57]
Nasopharyngeal cancer	Methylation	MSP, −220 bp to −14 bp MSP, −220 to −14 bp	61 % (22/36) 59 % (31/53)	[80] [81]
Non-small cell lung cancer	Reduces expression Methylation Mutation	IHC MSP, −220 to −14 bp MSP, −220 to −14 bp MSP, −220 to −14 bp/IHC MSP −195 to −99 bpm MSP, −220 to −14 bp MSP, −220 to −14 bp	39 % (16/41) 19 % (7/37) 10 % (2/20) 15 % (3/20)/39 % (69/157) 32.4 % (100/308) (serum) 10 % (16/165) 3.1 % (1/32) 6 % 3/53	[63] [42] [74] [53] [47] [52] [63] [44]
Esophageal cancer	Methylation Copy number loss	MSP, −163 to −8 bp MSP, −227 to −86 bp bisulfite pyrosequencing qPCR	16.3 % (7/43) 24 % (9/38) 31 % (18/58) 59 % (16/27)	[46] [82] [45] [45]
Cervical cancer	Methylation	MSP, +168 to 318	12 % (2/14)	[59]
Hepatocellular cancer	Methylation	MSP, −225 to −85 bp	35 % (22/62)	[83]
Biliary tract carcinoma	Methylation	MSP, −9 to +98 bp	16 % (6/37)	[84]
Oral squamous cell cancer	Methylation	MSP, −220 bp −14 bp	31 % (4/13) 34.7 % (17/49)	[85] [86]
Cutaneous T-cell lymphoma	Methylation	CpG island microarray	19 % (5/28)	[87]
Head and neck cancer		COBRA MS-MLPA	30 % (16/54) 25 % (7/28)	[73] [88]
Endometrial cancer	Methylation	MSP, +168 to 318 bp	12 % (6/50)	[60]

IHC, immunohistochemistry; (MS)-MLPA, (methylation-specific) multiplex ligation-dependent probe amplification; MSP, methylation-specific PCR; COBRA, combined bisulfite restriction analysis

Additional studies to identify structural variations in the *CHFR* coding sequence led to the identification of three missense mutations in non-small cell lung cancer (NSCLC); all three were associated with a defective mitotic checkpoint [44]. Two mutations target the FHA and RING finger domain and the third is located in the cysteine-rich region (Fig. 2b). These mutations, however, were observed in only 3 out of 53 patients. Loss of the chromosomal region harboring *CHFR*, 12q24.33, occurs more frequently. In esophageal adenocarcinomas (EAC), *CHFR* DNA copy number loss appears to occur in 59 % (17/27) of esophageal cancers and is associated with reduced *CHFR* expression [45].

In most cancers, however, *CHFR* expression is reduced due to promoter CpG island methylation (Fig. 2a). The promoter region of *CHFR* contains a CpG island spanning −905 to +783 bp relative to the transcription start site. *CHFR* promoter CpG island methylation and subsequent transcriptional silencing was first described in esophageal cancer [46], of which 16.3 % (7/43) was hypermethylated while this was absent in adjacent normal tissues. Later it became clear that *CHFR* promoter CpG island methylation occurs in other cancers as well, among which CRC (24–53 %) and gastric cancer (35–52 %) (Table 1). In NSCLC, *CHFR* promoter CpG island methylation occurs in approximately 10–40 % of NSCLCs characterized by wild-type *EGFR* and *KRAS* in absence of *ALK* gene rearrangement, which implicates that *CHFR* promoter CpG island methylation occurs in a specific NSCLC subgroup [47].

Multiple studies have shown that *CHFR* promoter CpG island methylation can be detected not only in the primary cancers but also in blood (NSCLC) [47], stool (CRC) [48] and peritoneal fluid (gastric cancer) [49, 50]. This lends support to *CHFR* having promise as a diagnostic marker.

## *CHFR* promoter methylation is associated with a poor prognosis and increased sensitivity to microtubule inhibitors

### *CHFR* promoter CpG island methylation as prognostic marker

In recent years, it has become clear that *CHFR* promoter CpG island methylation is associated with a poor prognosis in multiple cancer types. In NSCLC, *CHFR* promoter CpG island methylation is associated with an increased risk of disease recurrence and poor survival [51–53]. In a series of 165 NSCLCs in which the *CHFR* promoter CpG island was methylated in 10 % of patients and *KRAS* and EGFR mutation were found in 8 % and 29 % of cases, *CHFR* promoter CpG island methylation was the only molecular alteration that was associated with a shorter survival (log-rank test, *P* = 0.0017) [52]. In colorectal cancer, an association between *CHFR* promoter CpG island methylation and poor prognosis has been reported in two independent studies. Tanake et al. [54] showed in a retrospective study of 82 resected high-risk stage II or III CRC that *CHFR* promoter CpG island methylation (assessed by pyrosequencing) was associated with a shorter recurrence free survival (log-rank test, *P* = 0.006) and a reduced overall survival (log-rank test, *P* = 0.07). We also recently showed that *CHFR* promoter CpG island methylation is an indicator of poor survival in stage II *BRAF* wild type microsatellite stable CRC (*n* = 66, *P* < 0.01, HR = 3.89, 95 % CI = 1.58–9.60) and validated these results in an independent prospective cohort study (*n* = 136, *P* = 0.07, HR = 2.11, 95 % CI = 0.95–4.59) (Cleven et al., submitted).

Together, these studies indicate that *CHFR* promoter CpG island methylation is an indicator of an aggressive phenotype characterized by a high risk of disease recurrence and a shorter overall survival. Testing for *CHFR* promoter CpG island methylation may help to select patients with a poor prognosis. Future studies are needed to investigate which treatment or screenings approaches will improve survival for patients with *CHFR* inactivated cancers.

### *CHFR* promoter CpG island methylation as predictor of taxane sensitivity

Although *CHFR* promoter CpG island methylation is associated with a poor prognosis, *CHFR* inactivation predisposes to an increased sensitivity to microtubule inhibitors (Table 2). Microtubule inhibitors such as docetaxel and paclitaxel disrupt normal microtubule dynamics during cell division by binding to the beta-tubulin subunits. This will lead to a failure of microtubule separation and apoptosis. As *CHFR* is able to block entry into prophase until chromosomal alignment is restored, *CHFR* inhibits the effect of taxanes. Accordingly, cells expressing *CHFR* are more viable upon treatment with microtubule inhibitors compared to cells not expressing *CHFR* [55].

**Table 2:** *CHFR* inactivation as prognostic and predictive marker

Cancer prognostic marker		Method	Ref
Lung cancer	Reduced *CHFR* expression associated with poor prognosis (*n* = 157) *CHFR* Promoter methylation associated with poor prognosis (*n* = 208)	IHC MSP	[53] [51]
Colorectal cancer	*CHFR* promoter methylation associated with poor prognosis in stage II MSS BRAF wt CRC (*n* = 66). Confirmed in second independent series (*n* = 136) *CHFR* promoter methylation associated with reduced recurrence-free and overall survival (*n* = 82)	MSP PS	[54]
Predictive marker			
Gastric cancer	*CHFR* promoter methylation associated with increased sensitivity to paclitaxel (cell lines *n* = 4) *CHFR* promoter methylation associated with increased sensitivity to paclitaxel (*n* = 12) No relationship between *CHFR* promoter methylation and sensitivity to docetaxel or paclitaxel (*n* = 41)	MSP MSP COBRA	[56] [57] [58]
Cervical cancer	*CHFR* promoter methylation associated with increased sensitivity to paclitaxel (cell lines, *n* = 6)	MSP	[59]
Oral squamous cell carcinomas	Silencing of *CHFR* with siRNA increases taxane sensitivity (cell lines)	siRNA	[85]
Lung cancer	*CHFR* promoter methylation associated with increased sensitivity to paclitaxel (*n* = 69 and 41) Unmethylated *CHFR* promoter associated with good response to EGFR TKIs (*n* = 179) Reduced *CHFR* expression predicts outcome to paclitaxel based therapy (*n* = 41)	MSP MSP IHC	[62, 63] [47] [63]
Endometrial cancer	*CHFR* promoter methylation associated with increased sensitivity to paclitaxel (cell lines, *n* = 6)	MSP	[60, 61]

IHC, immunohistochemistry; MSP, methylation-specific PCR; PS, pyrosequencing; siRNA short interference RNA; TKI, tyrosine kinase inhibitors

The association between *CHFR* expression and decreased sensitivity to microtubule inhibitors was first shown by Satoh et al. in gastric cancer cell lines [56]. Docetaxel or paclitaxel is a standard treatment option for gastric cancer, though not all patients will respond to this therapy. *CHFR* promoter CpG island methylation was hypothesized to be an important determinant of response to therapy. In a small study of 12 patients with advanced stage gastric cancer that received adjuvant paclitaxel, *CHFR* promoter CpG island methylation was associated with a better clinical response compared to cancers with unmethylated *CHFR* of which the majority showed progressive disease [57]. These results could, however, not be confirmed in a larger study of 41 gastric cancers in which promoter CpG island methylation was not associated with response to docetaxel or paclitaxel [58]. Of note, clinical response, however, was measured in metastatic lesions where the methylation status of *CHFR* was not assessed.

Stronger support for the potential role for *CHFR* promoter CpG island methylation in predicting response to microtubule inhibitors has been described in other cancer types. Cervical adenocarcinoma cell lines (*n* = 6) with *CHFR* promoter CpG island methylation, for instance, are sensitive to treatment with docetaxel and paclitaxel [59]. Treatment with 5-Aza-2′-deoxycytidine recovered *CHFR* expression and decreased the sensitivity to these agents immediately, an effect that was not observed for treatment with 5-fluorouracil, etoposide, cisplatin and doxorubicin. The same accounts for *CHFR* promoter CpG island methylation and treatment with paclitaxel in endometrial cancer cell lines [60, 61] and in NSCLCs [62].

Other preclinical evidence for *CHFR* as marker of taxane sensitivity comes from a recent retrospective study that analyzed *CHFR* inactivation and response to paclitaxel in metastatic NSCLC [63]. *CHFR* promoter CpG island methylation was assessed with MSP and validated by methylation microarray and nuclear expression of *CHFR* was analyzed by immunohistochemistry. Although *CHFR* promoter CpG island methylation was observed in only 1/32 (3.1 %) patients, 16/41 (37 %) patients showed reduced nuclear staining of *CHFR*, indicating the presence of a *CHFR* repressive event other than promoter CpG island methylation that still needs to be elucidated. In this study diminished nuclear *CHFR* expression was associated with a better response to therapy (19 % versus 52 % progression at first restaging, *P* = 0.033) and a prolonged overall survival (9.1 versus 5.1 months, HR 0.28, 95 % CI = 0.14–0.56) compared to patients with high *CHFR* nuclear expression.

Finally, in colorectal cancer, a recently reported preclinical study showed increased sensitivity to taxanes in colorectal cancer cell lines both *in vitro* and *in vivo*. The correlation between *CHFR* expression and resistance to docetaxel was statistically significant (*P* = 0.033), with a 20-fold increase in median IC50 for cell lines that had measurable *CHFR* expression versus silenced cell lines [64].

Although randomized prospective clinical trials are needed before implementation into clinical practice, these studies together strongly support the evidence for *CHFR* inactivation as marker of taxane sensitivity.

## Conclusion

In the last decade, a substantial number of studies have been performed to investigate *CHFR* inactivation, usually due to promoter CpG island methylation, as biomarker to predict prognosis and response to microtubule inhibitors in a diversity of cancers. There is compelling evidence that reduced *CHFR* expression is a promising biomarker that can improve the management of multiple tumor types.

The clinical impact of *CHFR* promoter CpG island methylation as prognostic marker will be in the selection of patients with an aggressive phenotype. In stage II CRC, *CHFR* promoter CpG island methylation can help to identify patients with a worse prognosis that might benefit from adjuvant therapy. The same accounts for *CHFR* inactivation as predictive marker of taxane sensitivity where *CHFR* inactivation can help to select patients for taxane treatment. Furthermore, these results can be a rationale for studying the effect of taxane treatment in cancers with *CHFR* inactivation. A prospective trial to test this hypothesis in CRC is presently ongoing.

The clinical value of any biomarker, however, depends on the accuracy of the test. The majority of candidate biomarkers reported in literature do not reach clinical use mostly because they fail to pass the validation phase. This can be explained by intra- and inter-tumor heterogeneity, a technical inability to consistently verify the presence of the biomarker in patient’s material, and the lack of specificity for a particular disease. In most studies, *CHFR* promoter CpG island methylation is assessed by methylation-specific PCR (MSP). MSP is a very sensitive qualitative method that is able to detect aberrant methylation in minute amounts of DNA [65]. One important aspect of the technique is the region selected to be analyzed since not all regions within the CpG island have biological and clinical relevance [66]. In the literature, however, different locations within the promoter CpG island of *CHFR* have been analyzed (Table 1), which makes it difficult to compare results. CpG methylation within region −240 to −73 bp relative to the transcription start site of *CHFR* has shown to result in gene silencing [46] and is therefore proposed as core region of promoter methylation [67].

One of the technical challenges of MSP is a false positive result due to inadequacy of bisulfite treatment and mispriming especially when nested PCR or high numbers of PCR cycles are used [65]. Several alternative methods are available among which pyrosequencing [68]. Pyrosequencing permits a quantitative methylation analysis with single nucleotide resolution of the amplified region but also relies on amplification of bisulfite-converted DNA. One of the challenges of pyrosequencing, however, is that the technique requires a numeric cutoff value to define a positive methylation status, which is difficult in a clinical setting that is dependent upon biopsy specimens that are usually small and do not allow correction for tumor heterogeneity or involvement of normal tissues. The same accounts for other quantitative (q)MSP techniques.

Besides MSP, pyrosequencing and qMSP, recently also ultra-deep next-generation-based bisulfite sequencing has become available. Future studies are needed to evaluate the performance of the different techniques in assessing *CHFR* methylation status in a clinical setting. Since all associations between *CHFR* promoter methylation and clinical variables (Table 2) until now are consistently found with MSP, at present MSP is the more promising method of choice.

Furthermore, large, independent cohort studies and clinical trials are needed to validate the prognostic and predictive value of *CHFR* inactivation. These trials will need to be compared to established clinical markers such as Tumor-Node-Metastasis (TNM) classification system. Simon et al. have proposed a less time consuming design in which archival material of prospective trials is used to investigate the performance of a single biomarker [69].

In conclusion, the combination of the crucial role of *CHFR* in mitotic checkpoint control and a clear prognostic and predictive power highlights the clinical potential of *CHFR* as biomarker. Although work is still in progress, currently available results all point into the same direction and make *CHFR* inactivation, mostly due to promoter CpG island methylation, a biomarker with great potential and the development of clinical trials to validate its predictive and prognostic value, a priority.
